# The Colour Problem in Hospital Life

**Published:** 1914-07-04

**Authors:** 


### THE COLOUR PROBLEM IN HOSPITAL LIFE.

### Coloured Students: Factors in the Controversy.

By AN OFFICER OF THE LONDON HOSPITAL.

The question as to the status and opportunities to be afforded to coloured students in this country is by no means an easy one, nor is it likely to be settled in the immediate future. In the hospital world there are records of coloured students playing in hospital matches at least thirty years ago, and in those days, as money in the medical colleges was very scarce, these students were welcomed by the Deans and Wardens. This question is warmly discussed not only by the authorities, but also by students of the Bar and of the Universities, as well as at the London hospitals, though we have not heard that it is the cause of any acute controversy in provincial hospitals. In hospital life there are students, patients, subscribers, and the general public to be considered, besides the staff and house committees.

From the students' point of view, one must admit that a policy of rigid exclusion would be most satisfactory, but then students' opinions are based upon prejudice and upon the fact that the coloured student most frequently lives apart from the Europeans, and his interests are not theirs. In this connection, too, the fact that when gathered together the coloured students speak in an unknown language gives impetus to the prejudices which used a few years ago to include even Welsh-speaking students.

From the point of view of hospital efficiency as dressers and clerks, and from their conduct in the wards, there is little to choose between the Indian, Chinese, or European student. Some are keen; most (if they have the chance) would prefer an easier existence; but the coloured students as a whole are not less trustworthy, less punctual, or less keen upon their work.

In considering the matter from the patients' point of view, there are really several aspects depending upon the sex and upon the personality of particular patients. For instance, to some people a black attendant who comes to them with the intimacy of a medical man is an abhorrent idea. To others the novelty is distinctly interesting, and it must be admitted that many coloured practitioners do exceedingly well in practice in working-class neighbourhoods. With regard to the question as to whether black students are acceptable to our female patients, the objection often arises with the male relatives of the patients rather than from the patients themselves; and when one remembers the critical atmosphere and vigilance that are always present in every ward, there is very little likelihood of any abuse occurring in this direction, heavy though it looms in some imaginations.

From the point of view of hospital staffs there is little to be gained by a large proportion of coloured students, inasmuch as the great majority of them return to their own country, and so they form no outposts from which cases would reach the staff later in consulting practice. Time devoted to teaching them brings no tangible return, whereas the gratitude of the other students is reflected in the prosperity of the physician's or surgeon's consulting private practice.

In the mind of the public at large there is a body of opinion quite tolerant of coloured races which would view with strong disfavour any policy of exclusion, and it is to the public at large that the hospitals make their great appeal. But public opinion would be very much against any appointment of coloured students who would have under their control sisters and nurses and students, and house appointments cannot be offered as an inducement to attract these students.

This question as to the admission of coloured students, involving, as it does, not only Indians, but Chinese, Japanese, and Malay folk, cannot be settled by the decision of any one hospital committee. It is in essence an Imperial question, and as long as the India Office and other advisory boards show a wise selection in the candidates for a higher medical education, it is difficult to see how the doors which have been opened in the past can now, as a result of simple prejudice, be slammed to.

